# Down Regulation of KAI1/CD82 in Lymph Node Positive and Advanced T-Stage Group in Breast Cancer Patients

**DOI:** 10.31557/APJCP.2019.20.11.3321

**Published:** 2019

**Authors:** Thammineni Krishna Latha, Ankur Verma, Gaurav Kumar Thakur, Basudev Banerjee, Navneet Kaur, Usha Rani Singh, Sonal Sharma

**Affiliations:** 1 *Department of Biochemistry, *; 2 *Department of Pathology, *; 3 *Department of Surgery, University College of Medical Sciences and GTB Hospital , University of Delhi, Dilshad Garden, Delhi, India. *

**Keywords:** Breast cancer, lymph node metastasis, KAI-1/CD82

## Abstract

**Background:**

Metastasis represents a deadly aspect of any cancer including breast cancer, given its high prevalence; treatment of metastatic breast cancer remains a clinically unmet need, which necessitates the exploration of metastasis suppressor genes (MSGs). *KAI-1/CD82* is an important member of MSGs; the role of *KAI1* has been well explored in prostate cancer, however its role in breast cancer is not fully explored and in fact the results of breast cancer studies are contentious. Thus, the present study aimed to investigate expression of *KAI1* at both transcriptional and translational levels in the tissue of breast cancer patients and benign breast disease. Further, we analysed the relationship between expression levels of *KAI1* and clinicopathological parameters in breast cancer patients.

**Materials and Methods:**

*mRNA* expression was studied by Real time PCR and protein expression was analyzed by both Western blot and Immunohistochemistry.

**Results:**

The results of the study indicate that *KAI1* expression was remarkably decreased in breast cancer both at the gene and the protein levels (P < 0.05) compared to benign breast disease. In addition, *KAI1* expression levels were strongly associated with axillary lymph node status and advanced T stage (p < 0.05), however no association was found with tumor grade, age, menopausal status and receptor status like ER, PR and Her2.

**Conclusion:**

Low expression of *KAI1* might be helpful for predicting the lymph node metastasis and T staging, thus predicts malignant prognosis of breast cancer.

## Introduction

Despite of the extensive progress in the screening methods, early diagnosis and myriad advancements in the treatment of breast cancer, it still remains the most predominant cause of cancer associated mortality in women globally (Gucalp et al., 2014). Metastasis continues to be the most lethal and ubiquitous aspect of several cancers including breast cancer and results in the failure of direct treatment. The survival rates vary widely for localized breast cancer and metastatic breast cancer which is nearly 90% for the former, while only dismal 20% for the later (Mukharjee and Zhao, 2013). One of the basic premises for primary tumor development is the crosstalk between oncogenes and tumor suppressors, likewise, metastasis also requires the interplay between metastasis promoting genes and suppressing genes (Zhao et al., 2015). In order to disseminate the cancer cells to secondary sites at distant organs, a cascade of events (angiogenesis, intravasation, extravasation, and growth at secondary sites) orchestrate metastasis, hence inhibition of any of these steps might suppress the metastasis. Nowadays focus of cancer research has shifted from exploring the mechanism behind metastatic paradigm to nip the metastatic process in bud itself, keeping in view that inhibition of metastasis could play a potential role in preventing cancer associated mortality. Thus, metastasis suppressor genes (*MSGs*) have provided new avenues for many cancer biologists, to work upon as they are responsible for the suppression of metastasis. 

*MSGs* are a class of genes that inhibit the metastasis process without inhibiting primary tumour formation (Yan et al., 2013). *Kai I* gene (*Kangai1/CD82*) belong to *MSG* family, originally identified in Prostate cancer cells, limiting its role not in prostate cancer only but also to various cancers such as lung cancer, pancreatic cancer, gastric cancer, bladder cancer, and breast cancer (Tonoli et al., 2005). The *KAI1* gene encodes an integral membrane protein (CD82) belongs to transmembrane 4 super family (TM4SF) and consists of four transmembrane domains and one large extracellular domain (Jee et al., 2006). In most metastatic cancers KAI1 expression is frequently down-regulated and it has recently been proposed that CD82 could be a promising biomarker for the prognosis of patients with malignant neoplasms (Malik et al., 2009) and for predicting the metastatic potential of several human cancers, including breast cancer (Steeg et al., 2003; (Zhong et al., 2016). Down regulation of *KAI1* has also been observed in brain metastasis of breast cancer progression at both transcriptional and translational levels (Stark et al., 2005).

Various clinicopathological characteristics are implicated in the prognosis, recurrence and survival in breast cancer; among them, tumor size, axillary lymph node involvement and extent of metastasis are one of several significant prognostic factors for breast cancer patients (Soerjomataram et al., 2008). In addition, expression of receptors like Oestrogen Receptor (ER), Progesterone Receptor (PR) and Her2/neu are well established prognostic and predictive factors for breast cancer which imparts credence to the significance of various clinic-pathological parameters in prognosis and prediction in response to several available therapeutic options in breast cancer (Singh et al., 2016).

Although extensive research over the years has shed light on the metastasis suppressing potential of *KAI1* in prostate cancer, limited studies exist in regard to breast cancer and particularly in relation to *KAI1* expression with clinicopathological features. Further, the lack of global consensus on the ambiguous role of *KAI1* expression in clinicopathological parameters especially tumor grade and receptor status of breast carcinoma has prompted us to investigate the expression levels of *KAI1* at both transcriptional and translational levels in the tissue of breast cancer patients and benign breast disease and to demystify the relationship between expression levels of *KAI1* and tumour stage, grade, axillary lymph node involvement, receptor status in breast cancer. 

## Materials and Methods

The study comprised of 100 histologically proven cases of breast cancer and 100 cases of benign breast disease. The samples were collected immediately after surgery from Department of Surgery, Guru Tej Bahadur (GTB) Hospital, (University of Delhi), Delhi, India. Breast tissue from tumour mass was obtained for the study. None of the patients received preoperative chemotherapy or radiation therapy before the operation. The study protocol was approved by Institutional Human Ethics Committee at University College of Medical Sciences (UCMS) and GTB Hospital, Delhi. Informed consent was obtained from all individual participants included in the study. 

The tissue biopsies were collected in Phosphate buffered saline (1X) and stored at -80°C until further use. The clinicopathological information on each case, including age, tumour size, nodal status, location, treatment, and metastasis, was obtained from patient records and the details are shown in the Table 1. The mRNA expression was studied by real time Polymerase Chain Reaction (RT-PCR) and Protein expression was evaluated by Western Blotting and Immunohistochemistry (IHC). 

*RNA isolation and cDNA synthesis*

Total RNA was extracted from the breast tissue using Trizol (Invitrogen Life Technologies, USA). The RNA concentration and purity was checked using Nano Drop device (Thermo Fisher, USA). Total RNA concentration of all specimens was more than 500 ng/µL and the 260/280 OD ratio was 1.8–2.0. Total RNA (1µg) was reverse transcribed to cDNA using iScript cDNA synthesis kit (Bio-Rad, USA) following manufacturer’s instructions. The RT (reverse transcription) reaction was performed at 25^o^C for 5 min, followed by heating at 46^o^C for 20 min and 95^o^C for 1min for inactivating reverse transcriptase.

*Quantitative analysis of KAI-1 mRNA by real-time PCR*

Real time PCR analysis for KAI-1 was performed using Eva green master mix as per the manufacturer’s instructions (Biorad, USA). Glyceraldehyde-3- phosphate dehydrogenase (GAPDH) gene was used as a reference gene to normalize the gene expression and the samples were run in duplicate along with no template control wells. In brief, 1ml of cDNA, 10µl of SSO fast Eva supermix and 1 μl each of forward and reverse primers were added and a final reaction volume of 20µl was made by adding Nuclease Free Water (NFW), subjected to the appropriate cycling conditions, which included: initial denaturation step at 95°C for 3 min, and forty cycles of 5 s at 95°C, 30 s for annealing step at 57°C for KAI1 primer and 62°C for GAPDH. Primer Sequences used for real time PCR are: KAI1 5’ CATGAATCGCCCTGAGGTCACCTA-3’ and 5’- GCCTGCACCTTCTCCATGCAGCCC-3’ and GAPDH 5’-AAATCAAGTGGGGCGATGCTG-3’, 5’-GCAGAGATGATGACCCTTTTG-3’. Analysis was done on CFX ConnectTM Real Time PCR Detection System (BioRad, USA). The fold change in gene expression was calculated using 2^-∆∆Ct^ method, in which ∆Ct was calculated using the difference between the Ct of target gene and reference gene; and then ∆∆Ct was calculated by the difference between ∆Ct of case and ∆Ct of control, which was then substituted in the formula 2^-∆∆Ct^. (Livak et al., 2001). 

*Analysis of KAI1 protein expression by western Blot*

Equal amounts of protein (20 µg) were loaded and separated by SDS-PAGE (12% gels) and subsequently transferred to PVDF membranes (Thermo Fischer Scientific, USA). Membranes were blocked for 1 h in 5% (w/v) dry milk, Tris-buffered saline, and 0.1% Tween 20. Primary antibodies (AHP 1709, Biorad, USA) as indicated were incubated with membranes for 2 h, and the membranes were washed three times for 5 min each time in Tris-buffered saline with 0.1% Tween 20. Subsequently, horseradish peroxidase-conjugated secondary antibodies were added for 1 h, and the membranes were washed three times in Tris-buffered saline with 0.1% Tween 20. Enhanced chemiluminescence reagent was used to detect membrane-bound protein by luminography. (My ECL Manager, Thermo Fischer Scientific). 

*Analysis of KAI-1 protein expression by immunohisto Chemistry (IHC)*

A total of 60 cases and 20 controls were included for the IHC analysis. The age, side, type of the tumor, grade, stage (pT), lymph node status and the presence or absence of in-situ component were studied in each case.

Construction of tissue microarray was accomplished using non prefabricated Paraffin blocks. Sections were taken on lysinated slides from the tissue microarray on which IHC was done for *KAI1*. Formalin fixed, paraffin embedded tissue sections were cut into 3-4 μm thick sections and deparaffinized with xylene three times each for 5 minutes, and rehydrated through a graded alcohol series(100%, 95%, 80%) each for 5 minutes at 95°C for 25 minutes in 0.01 M tris EDTA buffer (pH 9.0) for antigen retrieval. Sections were then incubated with 4% H_2_O_2_ for 30 minutes followed by 3 washings for 5 minutes each with TRIS buffer (pH 7.6) to block endogenous peroxidase activity and non specific binding. The sections were incubated with the rabbit polyclonal anti* KAI1* protein antibody (Proteintech, 10248-1-AP, USA) in a dilution of 1:200 at 4°C for overnight. They were treated with the HRP Polyclonal antibody for 20 minutes after 3 washes with TRIS buffer, each for 5 minutes and then incubated the sections for 20 minutes in pre-formed streptavidin-biotinylated peroxidase complex. Counterstaining was done for 30 seconds with Mayer’s haematoxylin followed by dehydration in alcohol grades, clearing in xylene and mounted in DPX after air drying. Sections from human tonsil were taken as positive control for *KAI1* protein expression. *KAI1* was scored based on cytoplasmic staining. A score of <5% was taken as negative, 6-50% as reduced and >51% as abundant.

A part from the KAI1 protein, ER, PR, Her-2 status for each case was studied immunohistochemically. For ER and PR scoring, the amount of positive staining was assessed as percentage of positive staining cells. Less than 1% positive cells were considered negative and more than 1% (score 1, 2 and 3) were taken as positive. For Her-2, a score of 3+ (strong complete membranous staining in >10% cells) was taken as positive. 

*Statistical analysis*

All the statistical analysis was done using SPSS-16 software program. Continuous data was summarized as mean ± SE, while categorical data in percentages %. Independent t-test was used to compare the expression levels of *KAI-1* in clinicopathological parameters between two groups and ANOVA was used to compare the expression levels of *KAI-1* in clinicopathological parameters between three groups or more. Correlations were tested by co-efficient of correlation (Pearson for parametric data and spearman for non parametric data). Comparisons were made between categorical groups by chi-square (χ^2^) test. For the association between qualitative parameters, chi-square/Fishers exact test was used. p<0.05 was considered as significant. 

**Table 1:** Clinicopathological Data of Breast Cancer Patients (n=100)

Characteristics	Number of patients (N or %)
Age (yrs)	
<40	44
>40	46
Tumor size (Cm)	
T1	8
T2	47
T3	29
T4	16
Grade	
1	39
2	42
3	16
Missing	3
Lymphnode metastasis	
Positive	57
negative	43
Estrogen Receptor	
Positive	58
Negative	37
Missing	5
Progesterone receptor	
Positive	47
Negative	45
Missing	8
Her2 receptor	
Positive	42
Negative	49
Missing	9
Menopausal status	
Pre menopause	57
Post menopause	43

**Figure 1:**
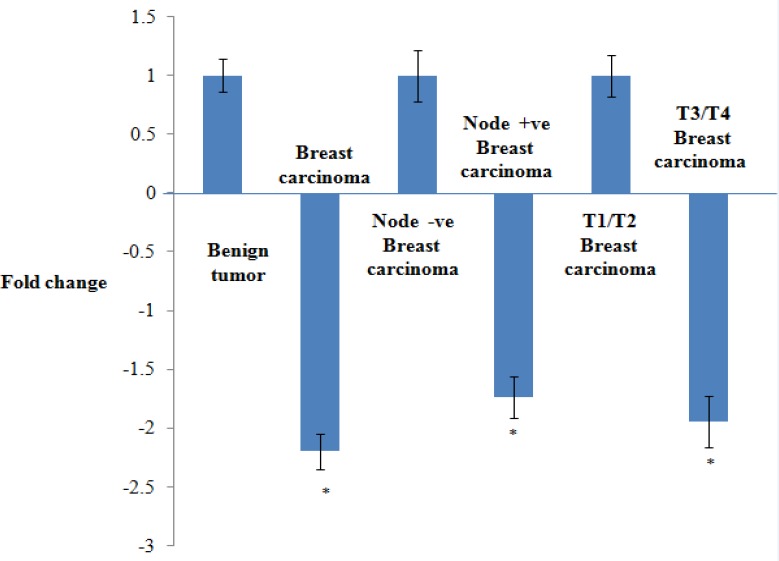
mRNA Expression Levels of KAI1in Cases and Controls and in Sub Groups of Cases. Benign breast disease group, Lymph node negative group and T1/T2 stage were taken as control and the fold change was considered as 1. mRNA expression of KAI1 is down regulated in breast carcinoma as compared to controls of benign breast disease. Further, KAI-1 expression is significantly down regulated in T3/T4 stage and node positive group of breast carcinoma with respect to T1/T2 stage and node negative groups of breast carcinoma respectively

*p<0.05 is considered as significant. Data was mentioned as SEM.

**Table 2:** The Relationship Between mRNA Expression of KAI1-1 and Clinicopathological Factors from Breast Cancer Patients (n=100)

Characteristic	(Mean ± SD) δCt KAI1	P value
Node		
negative	6.94± 1.47	0.006*
positive	7.74±1.37	
Age		
<40	7.5±1.52	0.4
>40	7.31±1.42	
≤ T2 (T1/T2)	6.95±1.34	0.001*
≥ T2 (T3/T4)	7.91±1.45	
Grade		
Grade 1 (G1)	7.26± 1.41	0.7
Grade 2 (G2)	7.52± 1.44	
Grade 3 (G3)	7.47±1.81	
ER receptor		
Negative	7.21±1.36	0.8
Positive	7.47±1.5	
Her2 receptor		
Negative	7.26±1.4	0.4
Positive	7.5±1.54	
PR receptor		
Negative	7.31±1.44	0.4
Positive	7.4±1.51	
Menopausal status		
Pre menopause	7.5±1.54	0.9
Post menopause	7.39±1.37	
Breast cancer	7.42±1.47	0.000*
Benign breast disease	6.26±1.57	

Data were presented in Mean ±SD; independent t–student test was applied to analyze the data, *p<0.05 was considered as significant.

**Figure 2:**
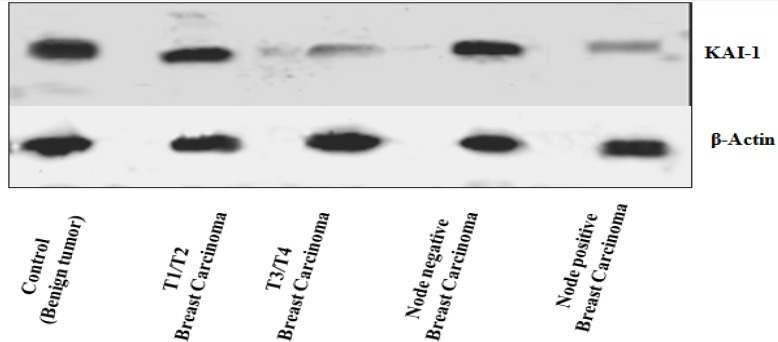
Western Blot Analysis of Protein Expression of KAI1and β Actin. Protein expression of KAI1 is lower in T3/T4 stage and node positive group (Lane2 & lane 4) compared to T1/T2 stage and node negative groups respectively

**Table 3:** Clinicopathological Data of Breast Cancer Patients Used for IHC Analysis (n=60)

Characteristics	No. of patients N (%)
Age	
<50	26 (43.4)
>50	34 (56.6)
Tumor size	
T1	7 (11.6)
T2	32 (53.3)
T3	15 (25)
T4	6 (10)
Grade	
1	18 (30)
2	30 (50)
3	12 (20)
Lymphnode metastasis	
Positive	34 (56.6)
Negative	26 (43.4)
Estrogen Receptor	
Positive	43 (71.7)
Negative	17 (28.3)
Progesterone receptor	
Positive	39 (65)
Negative	21 (35)
Her2 receptor	
Positive	9 (15)
Negative	51 (85)
Menopausal status	
Pre menopause	26 (43.3)
Post menopause	34 (56.6)

**Figure 3:**
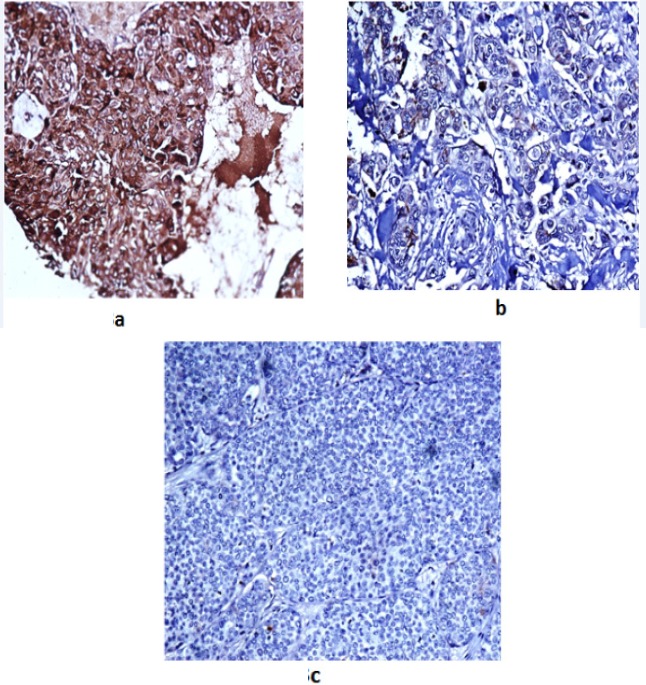
Representative Immunohistochemistry Staining for KAI1/CD82 Protein Expression in Paraffin-Embedded Breast Carcinoma. Fig 3a, strongly positive immunostaining i.e abundant expression of KAI1; Fig 3b, reduced immunostaining; Fig 3c, negative immunostaining

**Table 4:** The Relationship Between Protein Expression of KAI-1 Investigated by IHC and Clinicopathological Factors from Breast Cancer Patients (n=60)

Variables	Positive KAI1 expression N (%)	Reduced KAI1 expression N (%)	Negative KAI1 expression N (%)	p value
Menopausal status				
Pre-menopausal	5 (19.2)	16 (61.5)	5 (19.2)	
Menopausal	8 (23.5)	19 (55.8)	7 (20.5)	0.68
Grade				
I	5 (27.7)	10 (55.5)	3 (16.6)	0.64
II	4 (13.3)	20 (66.6)	6 (20)	
III	3 (25)	8 (66.6)	1 (8.3)	
Tumor size (Cm)				
T1	3 (42.8)	4 (57.1)	0	0.01*
T2	9 (28.1)	20 (62.5)	3 (9.3)	
T3	0	12 (80)	3 (20)	
T4	0	3 (50)	3 (50)	
Node				
Node Positive	0	22 (64.7)	12 (35.2)	<0.001*
Node Negative	13 (50)	13 (50)	0	
Luminal				
A	7 (24.1)	17 (58.6)	5 (17.2)	0.72
B	2 (14.2)	8 (57.1)	4 (28.5)	
Her2 positive	3 (33.3)	4 (44.4)	2 (22.2)	
Triple Negative	1 (12.5)	6 (75)	1 (12.5)	
ER receptor				
Positive	9 (20.9)	25 (58.1)	9 (20.9)	0.73
Negative	4 (23.5)	10 (58.8)	3 (17.6)	
PR receptor				
Positive	7 (17.9)	23 (59)	9 (23)	0.34
Negative	6 (28.5)	12 (57.1)	3 (14.2)	
Her2 receptor				
Positive	5 (21.7)	12 (52.1)	6 (26)	1
Negative	8 (21.6)	23 (62.1)	6 (16.2)	

## Results

In this study we have explored the expression levels of *KAI-1* in breast carcinoma and benign breast disease patients and investigated for association between *KAI-1* and clinicopathological parameters in the patients of breast cancer. A comparison analysis of gene expression was performed according to clinicopathological features like T stage, grade, lymph node metastasis, receptor status (ER, PR and HER2), menopausal status and age. Breast cancer patients were selected who had not received any preoperative chemotherapy as this might affect the *KAI-1 *expression levels being measured in the tumours. 

The distribution of patients’ details appears in Table 1. The age range for the study population was 25 to 75 years. Pre and post-menopausal patients comprised 57% and 43% of the population, respectively. Lymph node metastasis positive was found to be 57% of the study population and negative in 43% of population. Tumor grading was carried out in all patients: 39% had grade I tumours, 42% had grade II, 16% had grade III and data was missing for 3 patients. The presence of ER, PR and Her2 was determined in all patients: 58% were ER-positive and 37% were ER-negative and data was missing for 8 patients; 47% were PR positive, 45% were PR negative, data was missing for 8; 42% were found to be Her2 receptor positive, 49% were Her2 negative and data was missing for 9 patients. 

*mRNA expression of KAI-1 gene in BC cases and BBD controls*

Relative *mRNA* expression of *KAI-1* gene in tissue of BC cases and BBD controls was analyzed by the ∆∆Ct method and the expression levels were compared with several established clinicopathologic prognostic variables in breast cancer cases. The relationship between *KAI-1 mRNA *and clinicopathological features of breast cancer has been summed up in Table 2. In this study, the mRNA level of *KAI-1* was significantly reduced in BC cases with respect to BBD and 2.2 folds down regulation were reported in BC cases. Additionally, aberrant expression of mRNA has been noticed in BC patients with lymph node metastasis group (node positive) as compared to node negative group and the difference between these two groups has reached statistically significant and observed 1.7 folds down regulation of *KAI-1 mRNA *expression in node positive BC cases. Further,* KAI-1* expression was reduced in larger tumor size (T3/T4) when referred to smaller tumor size (T1/T2), which was found to be statistically significant and shows 1.9 folds down regulation in the *mRNA* level of *KAI-1* in larger tumor size (T3/T4 group) (Figure 1). Notably, there were no apparent associations with other prognostic variables such as age, grade, menopausal status and receptor status (ER, PR and Her2 status) (Table 2). 

*Protein expression levels of KAI-1 as determined by Western blot and IHC*

Western blot analysis has shown lower expression of KAI-1 in BC cases as compared to BBD. Further, the mRNA and protein levels of* KAI-1* detected by real-time RT-PCR, Western blot and IHC were consistent with each other, from which it can be implicated that the reduced protein expression levels might derived from reduced transcription of these genes. *KAI-1* demonstrated to have considerably lower in node positive group and larger T size group as compared to node negative group and smaller T size (T1/T2) group respectively (Figure 2). β-Actin has been used as a reference protein (Figure 2).

*Protein expression detected by IHC*

IHC has been performed on a total of 60 cases and 20 cases of fibro adenoma controls. The relationship between KAI1 protein and clinicopathological features of breast cancer has been summed up in Table 4. *KAI1* expression was found to be reduced in 35/60 (58.3%) cases and negative in 12/60 (20%) cases. Fig 3 (a, b and c) shows abundant, reduced, and negative expression respectively. Further, it was found that expression of *KAI1* was abundant (>51%) in all the controls. The details of the patients have been shown in the table 3. The age of the patients included in the study ranged from 30 years to 75 years with the mean age being 51. There was no significant association between KAI1 expression and age of the patient (p value- 0.79). Out of 60 cases, 26 were pre-menopausal and 34 were menopausal and we did not find any significant association between* KAI1* expression and menopausal status of the patients (p value- 0.68). Tumor staging was done according to the AJCC classification. Majority of the cases 32/60 (53.33%) were in T2 stage. In T1 stage 4/7 (57.1%) cases, in T2 stage 20/32 (62.5%) cases, in T3 stage 12/15 (80%) cases and in T4 stage 3/6 (50%) cases expression of *KAI1* was reduced. A significant association between *KAI1* expression and stage of tumor was observed (p value- 0.01). Lymph nodes were positive in 34/60 (56.66%) while negative in 26/60 (43.33%). In 22/34 (64.7%) lymph node positive cases and in 13/26 (50%) lymph node negative cases expression of *KAI*1 was reduced and* KAI1 *expression was significantly associated with lymph node status (p value- <0.001). All the cases were graded using the Nottingham modification of Bloom Richardson system. Among 60 cases, 18 were Grade I, 30 were grade II and 12 were grade III and no association was found between *KAI1* expression and grade of tumor (p value- 0.64). Based on molecular subtypes, 29/60 (48.33%) were found to be luminal A subtype and 14/60 (23.33%) were of luminal B subtype and 8 cases of Triple negative subtype and we reported that *KAI1* expression was not associated with molecular subtype (p value-0.72). The Hormone Receptors (ER/PR) were studied and scored as per guidelines. They were labelled negative for score 0 and positive for score 1+/2+/3+. ER was found to be positive in 43/60 (71.66%) cases and negative in 17/60 (28.33%) cases and no significant association was found between *KAI1* expression and ER status (p value- 0.73). PR was found to be positive in 39/60 (65%) cases and negative in 21/60 (35%) cases and KAI1 expression did not found to differ between PR positive and negative (p value- 0.34). Her2 was found to be positive in 23/60 (38.33%) cases and negative in 37/60 (61.66%) cases and the results have shown no difference between Her2 positive and negative. 

## Discussion

A large number of the cancer associated mortality is attributed to metastasis, which leaves the medical management futile in breast cancer. Breast cancer is curable if detected at an early stage, whilst various intricacies leaves it incurable if detected at an advanced stage i.e. stage IV as malignant cells would have already been spread to distant sites. Despite of the drastic shift (substantial progress) in the diagnosis and treatment of breast cancer, 6-10% of patients are still detected with metastasis at the time of diagnosis, which culminates in grim survival rate, while the survival rate approaches nearly 100% for local breast cancer (Redig, and McAllister 2013; Jin and Mu 2015). This implies that metastasis is a strong determinant of the survival for breast cancer patients. Therefore, the discovery of molecules which can exclusively inhibit metastasis has heralded a new ray of hope to many cancer biologists and thus metastasis suppressors have received the most attention from revitalised research across the globe.

*KAI-1* (Kangai1 means anti cancer in Chinese) is one of the important member of metastasis suppressor genes, capable of suppressing tumor metastasis, first identified in a prostate cancer. It is also termed as CD82, a lymphocyte cell surface protein implicated in T cell receptor activation (Dong et al., 1995). *KAI-1* interacts with a large number of proteins, including integrins, G-protein-coupled receptors, Src Kinase and epidermal growth factor receptor (EGFR), which are implicated in the array of cellular events, such as cell adhesion, migration, survival, and cell differentiation (Hemler et al., 2001). It has been demonstrated that low expression of *KAI1* is correlated with the aberrant cell adhesion to the ECM components, lost of cell-cell interactions, and acquired cell motility; which collectively influences the invasive and metastatic potential of the malignant cells (Jee et al., 2006).

Although numerous studies available on the role of *KAI-1* in breast cancer, no unanimity exists regarding the correlation of *KAI-1* with clinicopathological features in breast cancer. In addition, the role of this gene has not been much explored in breast cancer, particularly in context to Indian population (Singh et al., 2017). Therefore, the present study has undertaken to investigate the gene and protein level expression of *KAI-1 *in the tissues of breast cancer and benign breast disease and correlated with the clinicopathological parameters of breast cancer. Further, to the best of our knowledge, this study is the first to investigate *KAI-1* expression using three different techniques i.e. Real time PCR, Western blot and IHC in the patients of breast cancer and confirmed a consistency between the mRNA expression investigated by Real time PCR and protein expression by both Western blot and IHC.

In the present study, a significant ablated expression of *KAI-1* was detected in breast cancer samples over con-trol samples at both mRNA level and protein level. Consistent with this finding, previous studies Malik et al., (2009) and Zhong et al., (2016) shown the low expression of KAI-1 in the tissue of breast cancer as compared to normal samples. Similarly, a study by Yang et al., (2000) demonstrated significantly high expression levels of *KAI-1* in normal breast tissues and benign breast tumours than in breast cancer.

Breast cancer is typically categorised in stages based on American Joint Committee on Cancer (AJCC) TNM classification, (Tumor Nodes Metastasis) which recommends the tumor size (T), presence of metastatic lymph nodes (N) and metastasis (M) are the fundamental determinants of staging (Kasangian et al., 2017). Axillary lymph node status is one of the most vital prognostic factors and lymph node involvement is associated with high risk of distant metastasis, regional recurrence, and poor survival in breast carcinoma, where as patients with lymph node negative have favourable prognosis (Howland et al., 2013; Dings et al., 2013). In the current study, we have observed an attenuated expression of *KAI-1 *in patients of breast cancer with axillary lymph node metastasis (ALNM) with corresponding to the group of breast cancer without ALNM. This finding is in accordance with the study by Zhang et al., (2012) who demonstrated that the *KAI-1* expression was down-regulated in breast cancer tissues and the patients with LNM shown decreased *KAI-1* expression as compared to the patients without LNM (Zhang et al., 2012). This might be attributed to the* KAI1* mediated stabilization of E-cadherin and β-catenin complex at the cell membrane which prevents dissemination of malignant cells from the primary tumor and thus limiting metastasis (Abe et al., 2008). Aberrant expression of *KAI-1* may trigger the tumor cells to loosen their homophilic cell adhesion, enabling them to dislodge from the primary tumour nest and intrude local tissues, and subsequently gaining access to blood stream and the lymphatic system (Chigita et al., 2012). 

Moreover, the expression levels of *KAI-1* were significantly attenuated in T3/T4 category with corresponding to T1/T2 category in our study (p<0.05). Our finding of low expression of *KAI-1* in T3/T4 category is in line with previous studies on breast cancer where they observed lower expression of *KAI-1* in advanced T category in comparison of early T category (Singh et al., 2016) and significantly higher transcription levels of KAI-1 in early stage tumours (TNM1) compared with late stage tumours (TNM2, 3 and 4 (Malik et al., 2000). Similar conclusions were derived on other cancers, for instance Guo et al (2015) established a significant negative correlation between the *KAI-1 mRNA* expression and TNM stage in gastric cancer patients and Zhuo et al also reported a negative association between *KAI-1* expression and clinical stage in cervical cancer patients (p<0.05) (Guo et al., 2015; Zhuo et al., 2015). This indicates that diminished expression of *KAI-1* plays a role in the progression of breast cancer.

Further, most of the current literature has concluded an inverse correlation between* KAI-1* expression and the severity of tumor, in other words, lower expression of *KAI-1* is associated with poor differentiation of the cancer cells in breast cancer (Chen et al., 2011; Zhang et al., 2012; Singh et al., 2016). However, in sharp contrast to these findings, we could not identify any such correlation between *KAI-1* expression and grade of the tumor which is similar to the finding by Malik et al where they did not find any association of *KAI-1* transcript level with the grade of the tumor (Malik et al., 2009). The inconsistencies in these results might be owing to differences in sample size, ethnicity, heterogeneous population, and methodological variability with respect to the antibodies and scoring categories etc. 

It is well established that the ERs and PRs implicated in the progression of breast cancer (Schuetz et al., 2011), further the lack of ERs and PRs has been associated with aggressive phenotype, lymph node involvement, tumor size and tumor grade (Ayadi et al., 2008). Few previous studies Huang et al., (2005); Christgen, (2008) and Christgen et al., (2009) shown that the ER-negative breast carcinomas frequently express *KAI-1*. Christgen et al., (2009) reported high frequency of *KAI-1* positive cases in both ER-negative primary tumors and ER negative metastases, which is paradoxical to the notion that *KAI-1* suppresses metastasis and stated that *KAI-1* may not be a useful marker to investigate invasive/ metastatic potential in breast cancer (Christgen et al., 2009). Conversely, a study by Zhong et al., (2016) observed that *KAI-1* expression is attenuated more often in ER- and PR-negative cases than that of positive cases, which suggested the loss of *KAI-1* is associated with poor prognosis in breast cancer (Zhong et al., 2016). On the contrary to the findings of the above mentioned studies, the results of our study shown that *KAI-1* expression has a insignificant association with ER, PR and Her2 status. 

This study has certain limitations such as failure to analyse the role of *KAI-1* in overall survival and in breast cancer recurrence, lack of patients who had distant metastasis, and heterogeneous population. In addition, *KAI-1* gene has chosen as the target of interest based on the literature/previous studies, not on the basis of micro array profiling, which might also a limitation of this study.

In conclusion, the aberrant expression of *KAI-1* was associated with lymph node metastasis, and advanced T stage in breast carcinoma. Evaluating *KAI-1* expression may help to predict the breast cancer patients with metastatic propensity, high aggressiveness and a poor prognosis as LNM is the primary conduit for distant metastasis in BC and associated with aggressive phenotype and poor prognosis. Further studies are required on a larger BC population size to confirm the clinical significance of *KAI-1* expression in breast cancer. 

## References

[B1] Abe M, Sugiura T, Takahashi M (2008). A novel functions of CD82/KAI-1 on E-cadherin-mediated homophilic cellular adhesion of cancer cells. Cancer Lett.

[B2] Ayadi L, Khabir A, Amouri H (2008). Correlation of HER-2 over-expression with clinico-pathological parameters in Tunisian breast carcinoma. World J Surg Oncol.

[B3] Chen X, Xu Z, Wang Y (2011). Recent advances in breast cancer metastasis suppressor 1. Int J Boimarkers.

[B4] Chigita S, Sugiura T, Abe M (2012). CD82 inhibits canonical Wnt signalling by controlling the cellular distribution of β-catenin in carcinoma cells. Int J Oncol.

[B5] Christgen M, Bruchhardt H, Ballmaier M (2008). KAI1/CD82 is a novel target of estrogen receptor mediated gene repression and down regulated in primary human breast cancer. Int J Cancer.

[B6] Christgen M, Christgen H, Heil C (2009). Expression of KAI1/CD82 in distant metastases from estrogen receptor-negative breast cancer. Cancer Sci.

[B7] Dings PJ, Elferink MA, Strobbe LJ, de Wilt JH (2013). The prognostic value of lymph node ratio in node positive breast cancer: a Dutch nationwide population-based study. Ann Surg Oncol.

[B9] Gucalp A, Gupta GP, Pilewskie ML, Sutton EJ, Norton L (2014). Advances in managing breast cancer: a clinical update. F1000 Prime Reports.

[B10] Guo J, Fan K, Xie L (2015). Effect and prognostic significance of the KAI1 gene in human gastric carcinoma. Oncol Lett.

[B11] Hemler ME (2001). Specific tetraspanin functions. J Cell Biol.

[B12] Howland NK, Driver TD, Sedrak MP (2013). Lymph node involvement in immunohistochemistry-based molecular classifications of breast cancer. J Surg Res.

[B13] Huang H, Groth J, Sossey-Alaoui K (2005). Aberrant expression of novel and previously described cell membrane markers in human breast cancer cell lines and tumors. Clin Cancer Res.

[B14] Jee BK1, Park KM, Surendran S (2006). KAI1/CD82 suppresses tumor invasion by MMP9 inactivation via TIMP1 up-regulation in the H1299 human lung carcinoma cell line. Biochem Biophys Res Commun.

[B15] Jin X P, Mu (2015). Targeting breast cancer metastasis. breast cancer: Basic Cli Res.

[B16] Kasangian AA, Gherardi G, Biagioli E 2017) The prognostic role of tumor size in early breast cancer in the era of molecular biology. PLoS One.

[B17] Livak KJ, Schmittgen TD (2001). Analysis of relative gene expression data using real-time quantitative PCR and the 2- ΔΔCT method. Methods.

[B18] Malik FA, Sanders AJ, Jones AD (2009). Transcriptional and translational modulation of KAI1expression of KAI1 expression in ductal carcinoma of the breast and the prognostic significance the breast and the prognostic significance. Int J Mol Med.

[B19] Mukherjee D, Zhao J (2013). The role of chemokine receptor CXCR4 in breast cancer metastasis. Am J Cancer Res.

[B20] Redig AJ, McAllister SS (2013). Breast cancer as a systemic disease: a view of metastasis. J Intern Med.

[B21] Schuetz F (2011). Adjuvant systemic therapy of breast cancer. Breast Care (Basel).

[B22] Singh R, Bhatt ML, Singh SP (2016). Expression levels of Tetraspanin KAI1/CD82 in breast cancer in North Indian females. Asian Pac J Cancer Prev.

[B23] Soerjomataram I, Louwman MW, Ribot JG (2008). An overview of prognostic factors for longterm survivors of breast cancer. Breast Cancer Res Treat.

[B24] Stark AM, Tongers K, Maass N, Mehdorn HM, Held-Feindt J (2005). Reduced metastasis-suppressor gene mRNA-expression in breast cancer brain metastases. J Cancer Res Clin Oncol.

[B25] Steeg PS, Ouatas T, Halverson D, Palmieri D, Salerno M (2003). Metastasis suppressor genes: basic biology and potential clinical use. Clin Breast Cancer.

[B26] Tonoli H, Barrett JC (2005). CD82 metastasis suppressor gene: a potential target for new therapeutics?. Trends Mol Med.

[B27] Yan J, Yang Q, Huang Q (2013). Metastasis suppressor genes. Histol Histopathol.

[B28] Yang X, Wei L, Tang C (2000). KAI1 protein is downregulated during the progression of human breast cancer. Clin Cancer Res.

[B29] Zhang T, Ren G, Zhang Z, Zhang R, Li Y (2012). The expression of tumor metastasis suppressor gene KAI1 and matrix metalloproteinase 2 in breast cancer tissues. Afr J Pharm Pharmacol.

[B30] Zhao M, Li Z, Qu H (2015). An evidence-based knowledge base of metastasis suppressors to identify key pathways relevant to cancer metastasis. Sci Rep.

[B31] Zhong J, Wu Y, Zhou K (2016). Combined expression of Ecadherin and KAI1 is associated with lymph node metastasis and poor prognosis in breast cancer. Int J Clin Exp Pathol.

[B32] Zhou XL, Wang M (2015). Expression levels of survivin, Bcl-2, and KAI1 proteins in cervical cancer and their correlation with metastasis. Genet Mol Res.

[B33] Zhu J, Miao C, Liu S (2017). Prognostic role of CD82/KAI1 in multiple human malignant neoplasms: a meta-analysis of 31 studies. OncoTargets Ther.

